# Global assessment of exposure to faecal contamination through drinking water based on a systematic review

**DOI:** 10.1111/tmi.12334

**Published:** 2014-05-08

**Authors:** Robert Bain, Ryan Cronk, Rifat Hossain, Sophie Bonjour, Kyle Onda, Jim Wright, Hong Yang, Tom Slaymaker, Paul Hunter, Annette Prüss-Ustün, Jamie Bartram

**Affiliations:** 1 The Water Institute, University of North CarolinaChapel Hill, NC, USA; 2 World Health OrganizationGeneva, Switzerland; 3 Department of Geography and Environment, University of SouthamptonSouthampton, UK; 4 WaterAid UKLondon, UK; 5 Norwich Medical School, University of East AngliaNorwich, UK

**Keywords:** water safety, *E. coli*, thermotolerant coliform, disease burden, drinking water

## Abstract

**Objectives:**

To estimate exposure to faecal contamination through drinking water as indicated by levels of *Escherichia coli* (*E. coli*) or thermotolerant coliform (TTC) in water sources.

**Methods:**

We estimated coverage of different types of drinking water source based on household surveys and censuses using multilevel modelling. Coverage data were combined with water quality studies that assessed *E. coli* or TTC including those identified by a systematic review (*n* = 345). Predictive models for the presence and level of contamination of drinking water sources were developed using random effects logistic regression and selected covariates. We assessed sensitivity of estimated exposure to study quality, indicator bacteria and separately considered nationally randomised surveys.

**Results:**

We estimate that 1.8 billion people globally use a source of drinking water which suffers from faecal contamination, of these 1.1 billion drink water that is of at least ‘moderate’ risk (>10 *E. coli* or TTC per 100 ml). Data from nationally randomised studies suggest that 10% of improved sources may be ‘high’ risk, containing at least 100 *E. coli* or TTC per 100 ml. Drinking water is found to be more often contaminated in rural areas (41%, CI: 31%–51%) than in urban areas (12%, CI: 8–18%), and contamination is most prevalent in Africa (53%, CI: 42%–63%) and South-East Asia (35%, CI: 24%–45%). Estimates were not sensitive to the exclusion of low quality studies or restriction to studies reporting *E. coli*.

**Conclusions:**

Microbial contamination is widespread and affects all water source types, including piped supplies. Global burden of disease estimates may have substantially understated the disease burden associated with inadequate water services.

## Introduction

Access to safe drinking water has long been a central aim of public health and international development policy. The Millennium Development Goals (MDGs) included target 7c to ‘halve by 2015 the proportion of the population without sustainable access to safe drinking water...’ (United Nations 2013). The World Health Organization (WHO) and UNICEF through their Joint Monitoring Programme (JMP) were tasked with monitoring progress against the MDG target and adopted an indicator, ‘use of an improved source’ (WHO/UNICEF 2013a). The indicator is based on a facility type classification with sources such as boreholes and piped supplies classed as improved and unprotected sources, such as uncovered dug wells, classed as unimproved (Table 1). In 2012, it was reported that the target had been met 5 years ahead of schedule (WHO/UNICEF 2012).

**Table 1:** Water quality ladder (WHO/UNICEF, 2012)

Source class	Source types
Piped on premises	Piped water connection located inside the user's dwelling, plot or yard
Other improved	Public taps or standpipes, tube wells or boreholes, protected dug wells, protected springs and rainwater collection
Other unimproved	Unprotected dug well, unprotected spring, cart with small tank or drum and bottled water
Surface water	Surface water (e.g. river, dam, lake, pond, stream, canal or irrigation channel)

Recognition of The Human Right to Water and Sanitation (UNCESCR 2010; United Nations 2010) has generated increased scrutiny of the progress that has been achieved during the MDG period. In particular, the improved source indicator has received criticism for not adequately reflecting safety (Godfrey *et al*. 2011; Bain *et al*. 2012; Onda *et al*. 2012), a limitation acknowledged by the JMP (WHO/UNICEF 2011). The WHO Guidelines for Drinking-Water Quality recommend that faecal indicator bacteria (FIB), preferably *E. coli* or alternatively thermotolerant coliform (TTC), should not be detectable in any 100 ml drinking water sample (WHO 2011). Yet numerous reports document faecal contamination of drinking water sources especially in low-income countries, including four of five nationally representative surveys commissioned by WHO and UNICEF (Bain *et al*. 2012). The JMP recognises that not all improved sources offer the same level of service and reports progress against a water quality ladder (Table 1; WHO/UNICEF 2012). Proposals for global monitoring of drinking water quality have been put forward by working groups commissioned by the JMP (WHO/UNICEF 2013a). Specifically, these call for the monitoring of two service levels: ‘basic’ and ‘intermediate’. The latter service level is defined by criteria including the absence of *E. coli* at levels above 10 per 100 ml.

Bottled water is classed as improved if the household has access to an improved source for cooking and washing (WHO/UNICEF 2006).

There is substantial evidence to demonstrate that improved sources of drinking water can contain faecal contamination. In a systematic review of microbial drinking water quality, many improved sources including piped water were found to be contaminated with *E. coli* or TTC (Bain *et al*. 2014). The review compared the relative safety of different types of water sources and assessed the effectiveness of the improved source metric, but did not report pooled estimates for different source types or regions. Earlier studies estimate that approximately 1.8 billion people are exposed to faecal contamination through drinking water (Onda *et al*. 2012; Wolf *et al*. 2013). These studies examined the presence of FIB, but not their extent and only reported global estimates.

The 2010 Global Burden of Disease study estimates for diarrhoeal disease are based on the assumption that improved sources present no risk to health (Lim *et al*. 2012). In this paper, evidence from literature concerning microbial contamination of different source types was used to estimate global population exposure to faecally contaminated water. The objective of this study was to estimate population exposure by source type and region. We estimated the proportion of the population drinking water from a source with greater than one and greater than ten TTC or *E. coli* per 100 ml using the database of studies from a systematic review (Bain *et al*. 2014).

## Methods

**Figure 1:**
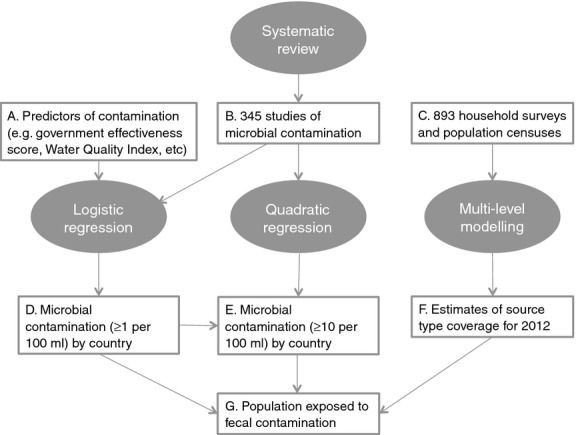
Analytical approaches used to estimate global exposure to unsafe drinking-water.

We combined estimates of the number of people using different types of water source in rural and urban areas with estimated levels of microbial contamination for each source type in a given country primarily based on data from a systematic review (Figure 1). As these estimates have been used to inform new estimates of the burden of diarrhoeal disease (Prüss-Ustün *et al*. 2014), we use WHO regions separated by income levels using the World Bank's classification (World Bank 2013).

### Water source coverage data

Household surveys and population censuses containing information on the proportion of households using different water sources (*n* = 893) were extracted from the JMP database (Figure 1, Box C; WHO/UNICEF 2013b). Following Table 1, improved sources included piped water to household, community piped water, protected dug wells, protected springs and boreholes. Unimproved categories included small tanker water, tanker water and surface water. Remaining sources were allocated to ‘other’ improved or unimproved sources and for the purposes of this analysis, assumed to be protected or unprotected groundwater.

We used multilevel modelling (Wolf *et al*. 2013) with a linear two-level model with a logit transformation of the dependent variable, time and WHO region as covariates and a random intercept and slope by country to estimate coverage (Figure 1, Box F). Multilevel modelling was applied separately by type of improved source and by urban/rural setting to estimate coverage in 2012. Likelihood ratio tests and the Akaike information criterion were used to select the models, and as these are relative measures, we also used residual plots to assess model fit. Estimates for the category ‘surface water’ were taken directly from the JMP (WHO/UNICEF 2012).

### Water quality data

A database of studies assessing water source contamination published between January 1990 and August 2013 was collated as part of a systematic review of faecal contamination in developing countries which extracted data on *E. coli* and TTC (Bain *et al*. 2014). The studies were restricted to those published in one of five languages (Chinese, English, French, Portuguese or Spanish) and separately reporting data by water source type. We incorporated additional piped water studies in China (*n* = 26) identified by, but not included in the review (Bain *et al*. 2014; Figure 1, Box B). These studies report quality prior to distribution and were included in our analysis to partially address underrepresentation compared with other large nations (e.g. India) as well as to increase the overall amount of water quality data. For this analysis, we used only those studies reporting presence or absence of FIB and/or FIB level classification (>1, 1–10, 10–100, >100 FIB per 100 ml). In total, 345 studies [319 from Bain *et al*. (2014)] were included in this analysis providing information on 133 460 water samples. Few water quality studies provide an adequate distinction between community piped and piped on premises as listed in Table 1. We therefore combined these categories (‘piped’) to match water quality data with coverage data. Tanker trucks and small carts were also combined.

### Predictive models

To estimate exposure to faecal contamination, we adapted an approach that has previously been used to extrapolate water quality on a global basis (Onda *et al*. 2012). We related data on water quality to covariates using logistic regression with random effects for each source type per study and binomial variance using *xtmelogit* functionality in Stata (Figure 1, Box A). This ensures that the estimated proportion lies between 0 and 1 and accounts for between study variance. We used the same seven covariates as Onda *et al*. (2012) to provide comparable estimates:

- Gross Domestic Product (GDP) per capita (World Bank 2011a)
- Government Effectiveness (GE) score (World Bank 2011b)
- Human Development Index (HDI) (World Bank 2011c)
- Water Quality Index (Srebotnjak *et al*. 2012)
- Annual aggregate precipitation (World Bank 2011a)
- % population attaining tertiary education (World Bank 2011d)
- Under-5 diarrhoeal morbidity rates (World Bank 2011a)

We combined covariates using principal component analysis (PCA) to derive uncorrelated synthetic variables; the first three of which were considered for inclusion in the water quality regression models based on a scree plot (Cattell 1966). As microbial water quality is known to differ substantially between rural and urban areas (Miranda *et al*. 2010; Bain *et al*. 2014), we also considered urban or rural setting in addition to publication year. Forward stepwise selection of principal components was used to create regression models for each water source type. We included the combination of covariates that resulted in the model with the greatest log likelihood, provided they were significantly different to the null model at the 5% level. Confidence intervals (95%) were determined using the standard errors of the model coefficients. Regressions were inverse-variance weighted as is common in meta-analysis (Borenstein 2009).

A subset of all water quality studies (*n* = 78) reported the proportion of samples containing >10 FIB per 100 ml. As these studies tend to report higher levels of contamination (Bain *et al*. 2014), we took a different approach to estimating exposure to >10 FIB per 100 ml. We developed an unweighted quadratic least squares regression model relating the proportion of samples containing FIB in a given study with the proportion exceeding 10 per 100 ml (Figure 1, Box E). We used this model to predict the population exposure to ≥10 per 100 ml for each water source type using our estimates of the population exposed to contamination.

In total, we fitted four regression models: piped, boreholes, unprotected groundwater (Figure 1, Box D) and the quadratic model linking presence of FIB to level of FIB (Figure 1, Box E). We used pooled estimates (i.e. no covariates) for two water source types: protected groundwater and tanker trucks. These models were then used to predict the proportion of contaminated sources, in rural and urban areas for all countries. We combined these predicted proportions with estimated coverage by source type in urban and rural areas by matching the estimates of the proportion of samples with ≥1 or ≥10 *E. coli* or TTC per 100 ml in each country. Estimates of the proportion and number of people (and their respective confidence intervals) were then calculated by summation (Figure 1, Box G).

### Sensitivity analysis

We investigated anticipated bias by restricting the analysis to studies reporting *E. coli* and studies with higher-quality ratings (>5 out of 13). Study quality ratings were based on 13 criteria as used in the systematic review (Table S1; Bain *et al*. 2014). We conducted a separate analysis for high-income regions using data for 12 countries in Europe available through the Protocol on Water and Health (UNECE 2013). As the number of samples has not been reported, we used fractional logistic regression (Onda *et al*. 2012).

## Results

### Population access by water source type

The proportion of the population in each region with access to different types of water source as calculated using multilevel modelling is shown in Table 2. The data sets used to estimate the proportion of country populations having access to specific types of source ranged from between 210 and 719 household surveys (Table S2). Residuals followed approximately normal distributions as illustrated by the plot for urban boreholes (Figure S1). Globally, the most commonly used sources of drinking water are piped supplies on premises (55.9%) and boreholes (17.0%). The use of piped sources off premises (e.g. public standpipes) is common in South-East Asia and Africa, the two regions where household piped connections are least widespread. The use of protected groundwater is common except in high-income countries in Europe and the Americas. The majority of users of unimproved sources live in Africa.

**Table 2:** Modelled proportion of the population using drinking water, by type of source and region in 2012

Setting	Region	Improved				Unimproved			Population (million)
		Piped on premises (%)	Piped off premises (%)	Boreholes (%)	Protected groundwater (%)	Unprotected groundwater (%)	Tanker (%)	Surface water (%)	
Urban	Africa	37.6	15.8	11.2	21.8	8.4	2.7	2.5	346
		Americas (HI)	97.3	0.9	0.9	0.9	0.0	0.0	0.0	291
		Americas (LMI)	93.7	0.9	0.9	2.0	1.6	0.8	0.1	478
		Eastern Mediterranean (HI)	79.3	1.3	0.8	17.2	1.1	0.0	0.3	40
		Eastern Mediterranean (LMI)	80.4	2.2	9.1	3.2	2.1	2.4	0.5	262
		Europe (HI)	99.5	0.1	0.1	0.3	0.0	0.0	0.0	350
		Europe (LMI)	92.8	1.9	1.3	3.1	0.6	0.1	0.1	289
		South-East Asia	47.2	12.4	23.3	11.9	4.2	0.6	0.4	628
		Western Pacific (HI)	99.0	0.4	0.3	0.3	0.1	0.0	0.0	188
		Western Pacific (LMI)	92.0	2.3	2.6	1.7	1.1	0.3	0.0	824
Rural	Africa	5.6	9.1	16.1	22.3	28.7	0.7	17.5	547
		Americas (HI)	95.2	0.2	1.5	1.4	1.6	0.2	0.0	63
		Americas (LMI)	63.4	3.0	5.8	9.8	10.5	1.0	6.5	124
		Eastern Mediterranean (HI)	69.3	2.7	2.7	20.6	4.5	0.2	0.0	7
		Eastern Mediterranean (LMI)	40.0	4.7	24.6	10.3	10.8	2.7	6.7	303
		Europe (HI)	98.8	0.1	0.3	0.7	0.0	0.0	0.0	107
		Europe (LMI)	54.1	8.1	8.9	20.4	3.7	1.5	3.2	159
		South-East Asia	12.7	12.5	48.5	12.6	11.8	0.3	1.7	1210
		Western Pacific (HI)	84.9	0.6	2.0	9.0	3.5	0.1	0.0	22
		Western Pacific (LMI)	40.4	2.9	23.1	19.3	11.6	0.4	2.4	811
Global		55.9	6.0	17.0	10.3	7.4	0.7	2.6	7050

HI, High income; LMI, low-middle income.

### Water quality regression model

Factor loadings for the first three principal components extracted from national data on predictors of source contamination are listed in Table 3. Together the first three components explain 82.2% of the variation. Regression models for piped water, boreholes and unprotected groundwater are shown in Table 4. Residuals followed approximately normal distributions as illustrated by the plot for boreholes (Figure S2). Tanker trucks (*n* = 4) and protected groundwater (*n* = 42) were not related to any of the first three principal components or any of the individual variables used in the principle components analysis (results not shown).

**Table 3:** Factor loadings from a principal component analysis of predictors of drinking water contamination for 195 countries

Variable	Principal component 1	Principal component 2	Principal component 3
Gross domestic product per capita	0.4210	0.1241	0.3595
Government effectiveness	0.4220	0.1646	0.3278
Human Development Index	0.4413	0.0985	0.2511
Aggregate precipitation	−0.1041	0.9143	−0.0190
Water Quality Index	0.3306	0.2149	−0.7968
Under five diarrhoea	−0.384	0.2188	0.2208
Tertiary Education	0.4270	−0.1336	−0.1273
Cumulative variation explained by principal components (%)	54.3	70.5	82.2

**Table 4:** Regression models used to determine population exposed to faecal contamination through drinking water

Variable	Type of water source		
	Piped [*β*, (SE)]	Borehole [*β*, (SE)]	Unprotected groundwater [*β*, (SE)]
Principal Component 1^1^	−0.881** (0.187)	–	–
Principal Component 2^1^	–	0.616** (0.168)	–
Principal Component 3^1^	–	–	0.884* (0.382)
Rural	1.737** (0.413)	–	–
Publication year	–	–	–
Constant	−2.000** (0.303)	−0.968** (0.209)	2.456** (0.386)
Obs. (no. of studies)	153	80	51
Wald chi (*P*-value)	31.34 (<0.0001)	13.52 (0.0002)	5.36 (0.0206)

* *P* < 0.05

** *P* < 0.0001.

1 For principal components see Table 3.

**Figure 2:**
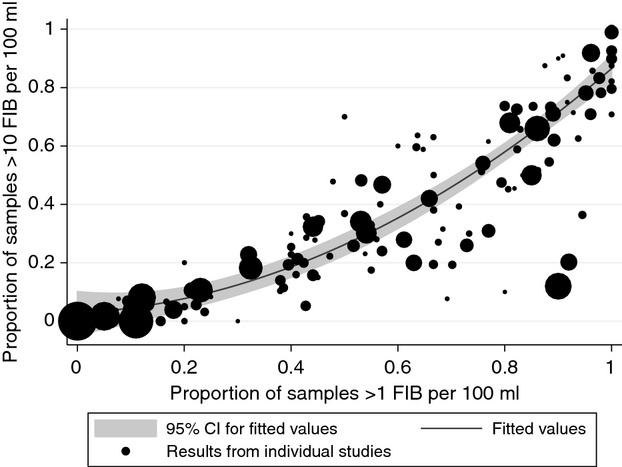
Relationship between proportion of samples contaminated with faecal indicator bacteria and the proportion with lev els greater than or equal to 10 per 100 ml, showing fitted quadratic model. Circles are proportional to the number of samples tested at a given type of water source within each study.]

Figure 2 illustrates the relationship between the proportion of samples found to be positive and the proportion containing at least 10 *E. coli* or TTC per 100 ml. This is based on 9495 samples from 78 studies. Although there are several outliers, we found that a quadratic model provides a reasonable fit to these data (*R*^2^ = 0.93; *n* = 151).

### Exposure estimates

Table 5 shows the predicted proportions of samples contaminated by region and source type as derived from the water quality regression model (Table 4). With the exception of high-income Europe, Americas and high-income Western Pacific, piped supplies are frequently contaminated in rural areas. Urban piped supplies were often contaminated in Africa, South-East Asia and low- and middle-income countries in the Eastern Mediterranean. Boreholes were generally more frequently contaminated than piped water with contamination ranging from 10% to 41%. The vast majority of unprotected groundwater sources contained FIB. We used pooled estimates from the null model for protected groundwater 0.56 (0.37–0.74) and tanker trucks 0.33 (0.12–0.64).

**Table 5:** Estimated proportion of samples contaminated by region and source type

Region	Piped		Boreholes	Unprotected groundwater
	Urban	Rural		
Africa	0.27 (0.15–0.42)	0.58 (0.41–0.71)	0.22 (0.15–0.31)	0.91 (0.82–0.96)
Americas (H)	0.00 (0.00–0.01)	0.01 (0.00–0.03)	0.21 (0.14–0.30)	0.97 (0.89–0.99)
Americas (LMI)	0.06 (0.03–0.11)	0.29 (0.20–0.41)	0.41 (0.30–0.53)	0.94 (0.86–0.97)
Eastern Mediterranean (HI)	0.03 (0.01–0.06)	0.14 (0.08–0.23)	0.10 (0.05–0.21)	0.97 (0.89–0.99)
Eastern Mediterranean (LMI)	0.20 (0.11–0.33)	0.51 (0.36–0.64)	0.18 (0.11–0.27)	0.89 (0.80–0.94)
Europe, (HI)	0.00 (0.00–0.01)	0.01 (0.00–0.05)	0.25 (0.18–0.34)	0.90 (0.81–0.95)
Europe, (LMI)	0.03 (0.02–0.07)	0.15 (0.09–0.24)	0.16 (0.10–0.26)	0.92 (0.83–0.96)
South-East Asia	0.11 (0.06–0.18)	0.35 (0.24–0.47)	0.32 (0.22–0.42)	0.78 (0.56–0.91)
Western Pacific (HI)	0.00 (0.00–0.01)	0.01 (0.00–0.05)	0.41 (0.30–0.52)	0.97 (0.89–0.99)
Western Pacific (LMI)	0.05 (0.03–0.10)	0.24 (0.16–0.35)	0.27 (0.18–0.37)	0.88 (0.78–0.93)

HI, High income; LMI, low-middle income.

**Figure 3:**
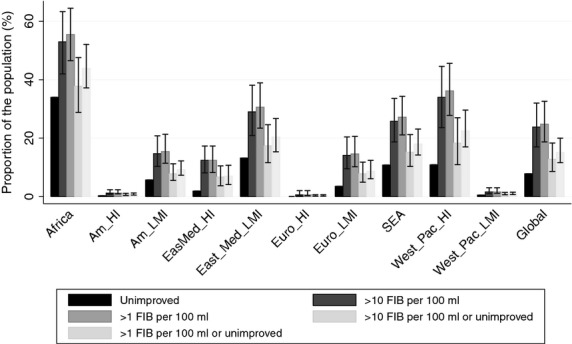
Proportion of the population exposed to faecally contaminated drinking water from improved sources or with access only to unimproved sources by region and globally for 2012. FIB: faecal indicator bacteria; HI: high income; LMI: low or middle income; Am: Americas; EasMed: Eastern Mediterranean; Euro: Europe; SEA: South-East Asia; West_Pac: western Pacific. Confidence intervals do not account for uncertainty in the relationship between presence (≥1 per 100 ml) and levels of contamination (≥10 per 100 ml) nor do they account for uncertainty in estimation from surveys and censuses.]

By combining the water quality data (Table 5) and coverage data (Table 2), we estimate exposure to faecal contamination by region and globally in Figure 3. Corresponding estimates for moderate to high levels of *E. coli* or TTC (>10 FIB per 100 ml) were determined using the relationship between presence and level of FIB illustrated in Figure 2.

The resulting model shows that the majority of people who use a contaminated water source live in South-East Asia (34%) or Africa (26%). An estimated 847 million [41% (CI: 31%–51%)] in rural areas and 224 million [12% (CI: 8%–18%)] in urban areas use contaminated water sources. The relative risk of contamination was greater for samples with higher levels of indicator bacteria: 6% (4–10%) of samples exceeded the 10 per 100 ml threshold in urban areas compared with 25% (18–34%) in rural areas.

Sensitivity of population exposure was investigated by restricting the included data to only high-quality studies and only those studies reporting *E. coli* (Table 6). Higher-quality studies predict greater contamination in high-income countries. This is consistent with data that we assessed from European countries provided to the United Nations Economic Commission for Europe (UNECE) as part of the Protocol on Water and Health (Table S2). Whereas the base estimates find that 0.6% of supplies in high-income European region are contaminated, the data from the protocol put the figure at 1.83% (CI = 1.15% to 2.13%).

**Table 6:** Sensitivity analysis of estimated proportion of regional and global population exposed to faecally contaminated drinking water in 2012

WHO Region, by income group	Proportion of the population (%)					
	Best estimate (*n* = 553)		Alternative estimate based on higher-quality studies only (*n* = 374)		Alternative estimate with studies on *E. coli* only (*n* = 230)	
	≥1 FIB per 100 ml	≥10 FIB per 100 ml	≥1 FIB per 100 ml	≥10 FIB per 100 ml	≥1 *E. coli* per 100 ml	≥10 *E. coli* per 100 ml
Africa	52.2	36.8	48.7	35.4	50.1	35.2
Americas (HI)	1.2	0.6	4.7	1.5	1.1	0.6
Americas (LMI)	14.6	7.7	16.9	8.1	12.9	6.7
Eastern Mediterranean (HI)	12.2	6.5	15.1	7.8	11.7	6.3
Eastern Mediterranean (LMI)	28.8	17.1	22.7	13.0	23.5	13.4
Europe (HI)	0.6	0.2	4.2	1.1	0.5	0.2
Europe (LMI)	14.0	7.7	16.5	7.9	11.6	6.3
South-East Asia	35.1	19.7	34.0	19.5	34.5	20.3
Western Pacific (HI)	1.5	0.9	5.7	1.9	1.6	0.9
Western Pacific (LMI)	23.8	12.8	25.4	13.8	21.0	12.0
Global	26.0	15.4	26.0	15.3	24.2	14.7

FIB, Faecal indicator bacteria; HI, high income; LMI, low or middle income.

We included all tanker truck studies even if low quality as there are only very few; *n* refers to the number of entries in the database.

## Discussion

### Exposure to faecal contamination

In 2012, we estimate that 1.9 billion (CI = 1.5–2.4) people used either an unimproved source or an improved source with faecal contamination. This global estimate is very close to previous reports (Onda *et al*. 2012; Wolf *et al*. 2013). Contamination is more frequent among some types of improved source, especially protected groundwater and rural piped supplies. Rural water sources are substantially more likely to be contaminated and generally suffer from higher levels of contamination than water sources in urban areas. The regions most affected by faecal contamination are Africa and South-East Asia; these are also the two regions with the lowest coverage of both improved water and sanitation (WHO/UNICEF 2012). Previous research suggests that water quality is adversely affected by inadequate sanitation (Howard *et al*. 2003).

Safety is one of several criteria, along with affordability and accessibility, which define acceptability of a water source in accordance with the Human Right to Water and Sanitation. Proposals for monitoring global access to drinking water after the MDGs have defined an ‘intermediate’ water service level which includes the requirement for supplies to contain fewer than 10 *E. coli* per 100 ml (WHO/UNICEF 2013a). We estimate that the number of people with an unimproved source or an improved source with ≥10 *E. coli* or TTC per 100 ml is 1.3 billion [95% CI: 1.0 – 1.6]. To create a baseline for the proposed ‘intermediate’ service level, these data would need to be combined with information on continuity of supply and accessibility. Intermittent piped supplies are more likely to be contaminated (Kumpel & Nelson 2013) and have been associated with adverse health outcomes (Klasen *et al*. 2012).

The prevalence of faecal contamination of drinking water recorded here has important implications for estimating the global burden of disease. The 2010 global burden of disease estimates (Lim *et al*. 2012) assumed that improved sources present no risk to health and that there are no health benefits associated with higher service levels such as a reliable piped supply on premises (Clasen 2014). This is questionable given the widespread faecal contamination of drinking water and the differences in the likelihood and extent of contamination between source types. These estimates may have substantially understated the diarrhoeal disease burden associated with inadequate water services.

We have not attempted to estimate the number of people actually consuming water that is faecally contaminated. Quality may deteriorate through unhygienic handling or ineffective storage, but may also be improved through active intervention or during storage (Wright *et al*. 2004). The extent of household water treatment has been reported (Rosa & Clasen 2010); however, effectiveness can be variable and practices may not be sustained (Brown & Clasen 2012). Furthermore, there is a hypothesised difference between contamination occurring in domestic and public domains (Cairncross *et al*. 1996). Whereas Vanderslice and Briscoe (1993) found that household contamination poses a lesser risk than contamination of the source, Trevett *et al*. (2004) argued that the risks may be at least as great.

### Predictive models

Piped water was more likely to be contaminated in rural areas. Water quality for piped supplies is correlated with the first principal component, suggesting that it is generally better in countries with higher GDP, HDI, GE and lower infant mortality. The second principal component is dominated by annual aggregate precipitation and is correlated to the quality of water from boreholes. Contamination events are known to coincide with rainfall (Howard *et al*. 2003); it is conceivable that this trend also occurs when comparisons are made between wet and arid countries. The proportion of samples from unprotected groundwater containing FIB is related to the third principal component and inversely correlated with the Water Quality Index; however, the correlation is relatively weak (*P* = 0.02). Quality of other source types was not related to the covariates which may reflect the limited data and extent of heterogeneity (Bain *et al*. 2014).

### Limitations

We were unable to estimate the coverage of rainwater by region, but this is known to be small globally, being the primary source of drinking water for 89 million people (1.2% of the global population) in 2010 (WHO/UNICEF 2012). The systematic review by Bain *et al*. (2014) showed that many rainwater samples are contaminated, albeit generally at lower levels than protected groundwater. Neither did we consider bottled nor sachet water in this analysis. An estimated 6% of the population primarily uses bottled water for drinking (WHO/UNICEF 2012), and as bottled waters are rarely contaminated (Bain *et al*. 2014), exposure may be overestimated. For high-income countries, water quality data from the Protocol on Water and Health show that our estimates may understate contamination.

With the exception of urban/rural setting, covariates included in the regression model were at the national level; if studies are clustered in particular areas of the country, they may not be representative for a given water source type. In addition, studies may not be representative of their region or country. For example, there may be few studies from conflict-affected countries, and studies in small towns may be rare compared with those in major cities. Moreover, our analysis is limited by the availability and quality of the covariates. For example, the Water Quality Index may not be reliable and has been removed from the Yale Environmental Performance Index due to concerns about the scarcity of the underlying Global Environmental Management System (GEMS) Water Programme data on environmental water quality (Srebotnjak *et al*. 2012). Greater predictive power may be gained using individual covariates rather than principal components, for example as applied to global estimates of the lack of wastewater treatment (Baum *et al*. 2013) as well as a broader selection of covariates. Furthermore, our confidence intervals do not fully reflect uncertainty including that arising from the coverage estimates.

Our estimates are largely based on studies that monitored water quality on one occasion. Due to variability in water quality, for example between seasons, these studies will tend to underestimate how many sources are faecally contaminated at some point in the year (Bain *et al*. 2014) and thus how many people are exposed to such contamination. We focused on two FIB that are known to suffer from a number of limitations but are widely used (Gleeson & Gray 1997). *E. coli* is a particularly sensitive indicator and will not survive as long as some pathogens such as cryptosporidium, especially after exposure to chlorine (WHO 2011); studies have found other FIB to be present in waters not containing *E. coli* (Sorlini *et al*. 2013). A comprehensive assessment of safety would take the presence of sanitary hazards and chemical contaminants including arsenic and fluoride into account. WHO promotes a proactive and comprehensive approach to risk management called Water Safety Plans (Davison *et al*. 2005; WHO 2011); their implementation can lead to improved water quality and public health outcomes in developed nations (Gunnarsdottir *et al*. 2012), indicating that even relatively well-managed utility piped systems do not present a negligible risk to health.

## Conclusions

Microbial contamination is widespread in lower- and middle-income countries and affects all water source types, including piped supplies. Drinking water is more likely to be contaminated in rural areas than urban areas, and faecal contamination was most prevalent in Africa and South-East Asia. The 2010 global burden of disease estimates (Lim *et al*. 2012) assume that improved sources present no risk to consumer. This is questionable given the widespread faecal contamination of drinking water outlined here, and thus, it is likely that disease estimates may have substantially understated the diarrhoeal disease burden associated with inadequate water services.

## Supporting Information

Figure S1 Histogram of residuals for logit access to urban boreholes.

Figure S2 Histogram of residuals for logit proportion of boreholes with detectable *E. coli* or TTC.

Table S1 . Quality criteria used to assess studies of microbial water quality in developing countries. **Table S2**. Number of surveys and censuses used in multilevel modeling. **Table S3**. Water quality data for high-income countries in Europe.
