# Supplementary material for: Global assessment of exposure to faecal contamination through drinking water based on a systematic review
Source: Trop Med Int Health. 2014 May 8;19(8):917–27. doi: 10.1111/tmi.12334 (PMC4255778; doi:10.1111/tmi.12334)
Supplement: Table S1 — . Quality criteria used to assess studies of microbial water quality in developing countries. Table S2. Number of surveys and censuses used in multilevel modeling. Table S3. Water quality data for high-income countries in Europe. [file tmi0019-0917-SD3.docx]

**Supplementary materials (Online-only)**

**Figure S1** Histogram of residuals for logit access to urban boreholes

**Figure S2.** Histogram of residuals for logit proportion of boreholes with detectable *E. coli* or TTC

**Table S1.** Quality criteria used to assess studies of microbial water quality in developing countries

| **Criterion** | **Question** |
| --- | --- |
| Selection  randomized | Was sampling randomized over a given study area or population? |
| Selection described | Do the authors describe how the water sources were chosen, including how either the types of water source or their users were selected? |
| Region specified | Does the study report the geographic region within the country where it was conducted? |
| Season reported | Were the seasons or periods during which sampling and/or inspections took place reported? |
| Representative | Was the study designed to provide representative picture water quality in a given area? |
| Quality control | Were quality control procedures specified? |
| Method described | Are well-defined methods of analysis described or referenced? |
| Point of sampling | Was the point at which water was sampled well-defined? (For example whether the water was collected from within a household storage container or directly from a water source) |
| Handling described^1^ | Are sample handling procedures described, including sample collection, method and duration of transport (if required) and incubation temperature? |
| Basic handling criteria^2^ | Does sample handling and processing meet the following criteria: transport on ice or between 2-8°C or field-testing, analysis within six hours of collection and specified incubation temperature (35 or 37 ±1°C for EC and 44.5 ± 1°C for TTC)? |
| Accredited lab | Was the microbial analysis conducted in an accredited laboratory? |
| Trained technician | Do the authors state whether trained technicians conducted the water quality assessments? |
| External review | Was the study subject to peer review? |

^1^ Studies referencing Standard Methods for the Examination of Water and Wastewater ([Rice et al. 2012](#_ENREF_43)) or other national or international standards are assumed to have followed the procedures outlined in these publications, i.e. that they stipulate quality control and handling procedures.

^2^ The criterion for analysis within 6 hours is more stringent that those specified in most standards, including the Standard Methods for the Examination of Water and Wastewater ([Rice et al. 2012](#_ENREF_43)) and the International Organization for Standardization (ISO) but is generally recommended for microbial water quality analysis ([WHO 2011](#_ENREF_10)).

Source: Bain et al. (2014)

**Table S2.** Number of surveys and censuses used in multilevel modeling

| **Type of source** | **# household surveys or censuses** | |
| --- | --- | --- |
|  | **Rural** | **Urban** |
| Piped on premises | 1034 | 1052 |
| Boreholes | 503 | 511 |
| Protected dug wells | 370 | 364 |
| Protected springs | 210 | 210 |
| Piped off premises | 714 | 719 |
| Unprotected groundwater | 572 | 570 |
| Tanker water | 428 | 437 |

**Table S3.** Water quality data for high-income countries in Europe

| **Country** | **Proportion of samples non-compliant^1^** (%) | **Year of reporting** |
| --- | --- | --- |
| Belgium (Flanders) | 0.16 | 2008 |
| Belgium (Wallonia) | 0.34 | 2008 |
| Croatia | 1.90 | 2009 |
| Czech Republic | 1.00 | 2008 |
| Estonia | 1.50 | 2009 |
| Finland | 0.05 | 2008 |
| France | 2.50 | 2009 |
| Germany | 0.10 | 2008 |
| Hungary | 1.10 | 2008 |
| Netherlands | 0.02 | 2008 |
| Norway | 8.60 | 2008 |
| Portugal | 2.19 | 2009 |
| Slovakia | 2.20 | 2007 |
| Pooled estimate for high-income Europe^2^ | 1.83 | - |

**^1^**To *E. coli* and *Enterococci* standards (<1 per 100 mL)

^2^This was based on a regression model with the first principal component (coefficient =-0.389, standard error = 0.152, p = 0.010).
